# Presentation and management of a case of rectal cancer complicated by perforation and necrotizing soft tissue infection

**DOI:** 10.1093/jscr/rjac318

**Published:** 2022-07-30

**Authors:** Chad E Cragle, James Schlenker, Ravi Moonka, Abigail Wiebusch, Vlad V Simianu

**Affiliations:** General and Thoracic Surgery, Virginia Mason Medical Center, Seattle, WA 98101, USA; Plastic and Reconstructive Surgery, Virginia Mason Medical Center, Seattle, WA 98101, USA; Colon and Rectal Surgery, Virginia Mason Medical Center, Seattle, WA 98101, USA; General and Thoracic Surgery, Virginia Mason Medical Center, Seattle, WA 98101, USA; Colon and Rectal Surgery, Virginia Mason Medical Center, Seattle, WA 98101, USA

## Abstract

A 68-year-old man presented with septic shock and severe perineal pain from a perforated low-rectal cancer causing a perineal necrotizing soft tissue infection. He underwent laparoscopic diverting colostomy and multiple surgical debridements resulting in extensive perineal and left leg wounds. A multidisciplinary rectal cancer team recommended against neoadjuvant chemoradiation or chemotherapy in his current state. He underwent up-front, urgent robotic-assisted abdominoperineal resection with immediate oblique rectus abdominus muscle flap closure. Final pathology demonstrated a T4N1b adenocarcinoma with negative resection margins. The patient subsequently underwent adjuvant chemotherapy. Now at over 18 months, he remains cancer free.

## INTRODUCTION

Treatment of rectal cancer has become increasingly complex and nuanced. Ideally, the management is guided by a multidisciplinary tumor board composed of surgeons, medical oncologists and radiation oncologists. The tumor board guides neoadjuvant, surgical and adjuvant treatments based on the patient’s unique presentation [1, 2]. Occasionally, rectal cancer may present with complications including obstruction, perforation and bleeding that create an acute or emergent illness that further challenges treatment of the underlying malignancy. Necrotizing soft tissue infections (NSTIs) are relatively rare, but life-threatening infections that occur infrequently as complications of perforated rectal cancer. We describe here the acute and definitive management of a case of rectal cancer which initially presented with a perineal NSTI.

## CASE REPORT

A 68-year-old man had been referred to colorectal surgery at our institution for a low rectal cancer and was scheduled for consultation and formal staging imaging with computed tomography (CT) and magnetic resonance imaging (MRI). However, he presented to an outside hospital acutely with worsening perianal pain. His physical exam was notable for exquisite tenderness of the perineum and left buttock, with an 8 cm necrotic eschar. He was in septic shock with a white blood cell count of 25 000/L, lactate of 4.2 mmol/L and soon required blood pressure support with norepinephrine. A CT scan was obtained showing extensive subcutaneous gas and edema within the perineum and buttocks. He was taken to the operating room for debridement and found to have a necrotizing infection of the perineum and perianal tissue. A concomitant laparoscopic diverting end sigmoid colostomy was performed.

**Figure 1:**
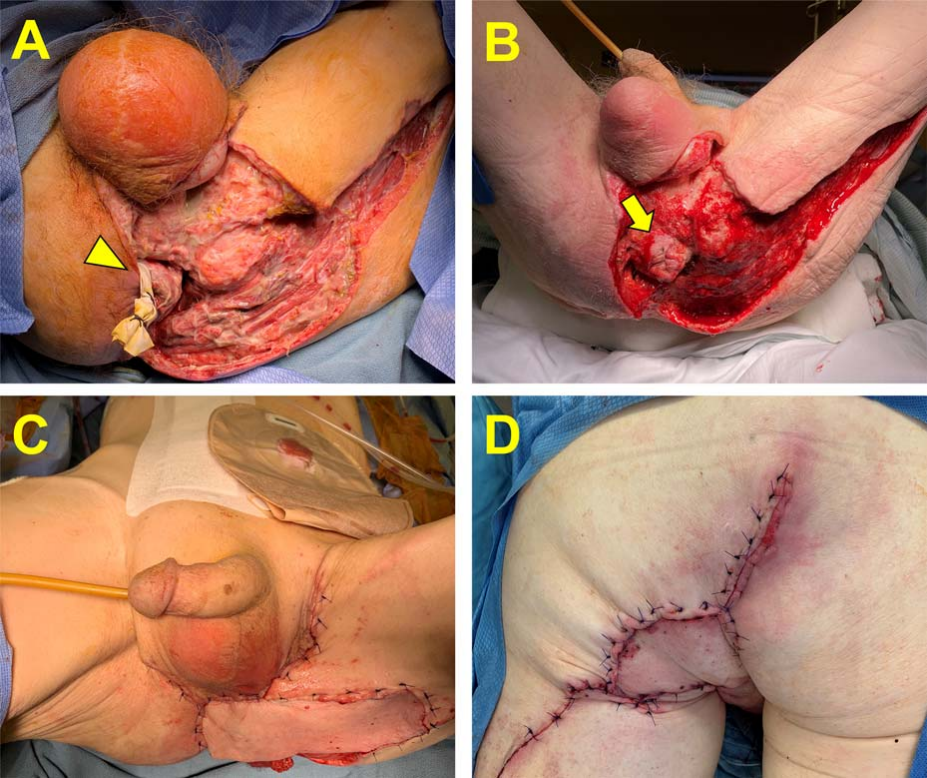
Management of the perineal wound. (**A**) Perineal wound upon transfer to our hospital following two initial debridements. A small skin bridge is encircled by a Penrose drain and connects the remaining perianal skin and the anus (arrowhead). (**B**) The perineal wound following complete infectious control and negative pressure wound vac therapy. Additional debridement was required and resulted in a free-floating anus (arrow). (**C** and **D**) The perineal wound following oncologic resection and flap reconstruction. Panel C is the anterior view, and Panel D is the posterior view.

He was subsequently transferred to our institution for definitive care which required two additional debridements before the infection was felt to be fully controlled. Attention was then turned to management of his extensive wound as well as treatment of his rectal cancer. The wound was initially managed with a negative pressure wound vac therapy (Fig. 1) and his CT staging imaging showed no evidence of distant metastatic disease. Our multidisciplinary tumor board discussed options for management of his rectal cancer. Considerations included neoadjuvant therapy with ongoing wound care versus definitive surgical resection. The decision was made to move forward with upfront surgical resection. The patient underwent a robotic-assisted extra-levator abdominoperineal resection and pelvic reconstruction utilizing a pedicled rectus abdominus myofasciocutaneous flap, scrotal advancement flap and rhomboid fasciocutaneous flap (Fig. 1).

Final pathology showed a pT4N1M0 moderately to poorly differentiated adenocarcinoma. Two of 13 perirectal nodes were positive for metastatic cancer. The mesorectum was graded as ‘incomplete’ due to the distal mesorectum having defects down to the muscularis and little bulk, secondary to prior debridement and perforation. Resection margins were negative. The patient subsequently underwent 6 months of adjuvant FOLFOX and remains cancer-free on active surveillance, now 18 months later.

## DISCUSSION

Optimal, contemporary treatment of rectal cancer begins with complete pre-treatment staging. For locally advanced rectal cancer (stage II and III), treatment is typically multimodal, and it is widely agreed that most patients should receive a combination of chemotherapy, chemoradiotherapy and surgical resection, though the sequence of these treatments continues to be debated [3–7]. However, when patients present with complications of their cancer, these treatment algorithms may need to be modified, increasing the importance of a multidisciplinary team.

Our patient presented in septic extremis and was fortunately stabilized after serial surgical debridements and aggressive resuscitation. There are few examples of the management of rectal cancer [8–10] or colon cancer [11–13] complicated by NSTI. These cases demonstrate the potentially fatal nature of this complication [8, 12]. They also highlight the importance of a staged approach to management, beginning with aggressive debridement, and following with oncologic management, if possible. However, there is a paucity of literature to guide the oncologic management of patients presenting with NSTI.

All rectal cancer patients at our institution are evaluated by a multidisciplinary tumor board. This was particularly important for this patient who was left with a large post-debridement wound that challenged the usual algorithm of care. To begin, he was not able to undergo our standard full staging work-up. A tissue biopsy was obtained and confirmed the diagnosis of adenocarcinoma, and he was able to undergo CT chest, abdomen, pelvis to screen for metastatic disease. However, a rectal cancer protocol MRI for locoregional tumor and nodal staging could not be obtained and the tumor board determined it would not have changed the recommendation for treatment. He was presumed to have a T4 tumor by NCCN staging criteria.

Determining the sequence of his cancer treatment was critical. The options of neoadjuvant radiotherapy and chemotherapy were considered. Neoadjuvant chemoradiotherapy has been shown to improve clearance of margins, reduce local recurrence and in some patients may even change the need for an abdominoperineal resection [4, 5]. In his case, the tumor board noted it would not change his need for abdominoperineal resection (APR), and there was concern with the potential morbidity of radiation on his extensive wounds. Neoadjuvant chemotherapy alone was also considered [6]. It was not expected that he would be able to complete the full standard course of chemotherapy without wound closure. Regarding wound management options, his wounds would not have been able to be closed without flap coverage, and the same tissue flaps would later be needed to close his APR defect. Furthermore, there was concern that skin grafting or prolonged negative pressure therapy would be unsuccessful if there was an underlying, untreated malignancy. For these reasons, upfront surgical resection was felt to be the best treatment option in his case.

After the patient recovered from surgery and was able to fully heal his wounds, he was recommended to complete 6 months of adjuvant chemotherapy with the goal of reducing his local and distant recurrence risk. He did successfully complete the full course of FOLFOX. Nonetheless, because of his final cancer stage and the circumstances of his resection, he is at a high risk for recurrence. If he does experience a local recurrence, he will still have the option of undergoing adjuvant chemoradiotherapy.
